# Rheophytic *Osmunda lancea* (Osmundaceae) exhibits large flexibility in the petiole

**DOI:** 10.1038/s41598-024-53406-4

**Published:** 2024-02-04

**Authors:** Masayuki Shiba, Tatsuya Fukuda

**Affiliations:** Graduate School of Integrative Science and Engineering, Tokyo City University, Tokyo, Japan

**Keywords:** Biophysics, Plant sciences, Stem cells

## Abstract

The riparian zone, found alongside rivers and streams, is a unique habitat characterized by its vulnerability to sudden floods following intense rainfall. To cope with these challenging conditions, a specific group of plants with linear and lanceolate lamina have adapted to thrive in these environments. Despite their unique ability to withstand the forceful water flow, the specific adaptive characteristics of the petioles, which support the lamina remain unknown. Our morphological, anatomical, and mechanical analyses on the petioles of *Osmunda lancea* (Osmundaceae) along the river and an inland sister species of *O. japonica* revealed that the petioles of *O. lancea* had a larger cell volume in subepidermal cortex and were more flexible than those of *O. japonica*.

## Introduction

Plant structure is subject to several functional constraints, the effects of which are dependent on the environmental stresses in the region in which the plant grows^1^. The occurrence of these environmental stresses varies in terms of both frequency and magnitude^2–4^. For instance, *Farfugium japonicum* (L.) Kitam. var. *japonicum* (Asteraceae) has a decreased lamina size to reduce the movement of the petiole so that the petiole does not break against strong winds in coastal areas^5^. Sudden flooding and rising water levels after heavy rainfall, are also environmental stressors that affect plants that grow alongside rivers^6^. Therefore, it has been suggested that these plants are capable of growing alongside rivers by acquiring distinct morphological features, such as linear and lanceolate leaves, as a means to mitigate the challenges posed by water flow stress ^7–9^. Plants with these morphological trait are have been observed to occur across a wide range of taxa ranging from bryophytes to angiosperms, indicating a parallel evolution^7^. Within the angiosperms, specifically in the case of *F*. *japonicum* var. *luchuense* (Masam.) Kitam., Usukura et al.^10^ observed a phenomenon known as leaf narrowing, which involved a reduction in the number of cells along the width of the leaf lamina. In addition, Shiba et al.^11^ reported that leaves of *Eurya japonica* Thunb. (Pentaphylacaceae) adapted to thrive in riparian zones by miniaturization rather than by leaf narrowing. From the above results, plants belonging to diverse taxonomic groups have undergone morphological modifications in their leaf lamina as an adaptive response to environmental stresses found in riverside habitats. In fact, Imaichi and Kato^12^ collected wild young sporophytes of *O. lancea* and *O. japonica* and studied their developmental stages in the experimental environment, and found that the morphology and anatomy of the young leaves of *O. lancea* had been similar to those of the leaves of field individuals actually exposed to water flow stress during development. However, the adaptation patterns for other organs, such as stems and petioles, have not yet been investigated. Certain studies have put forth the hypothesis that stems and petioles of plant groups along the river have developed flexibility as an adaptation to mitigate the impact of river water flow^7,12^. Therefore, elucidating the specific adaptation patterns exhibited by the stems and petioles of plant groups along the river would be of great interest.

**Figure 1:**
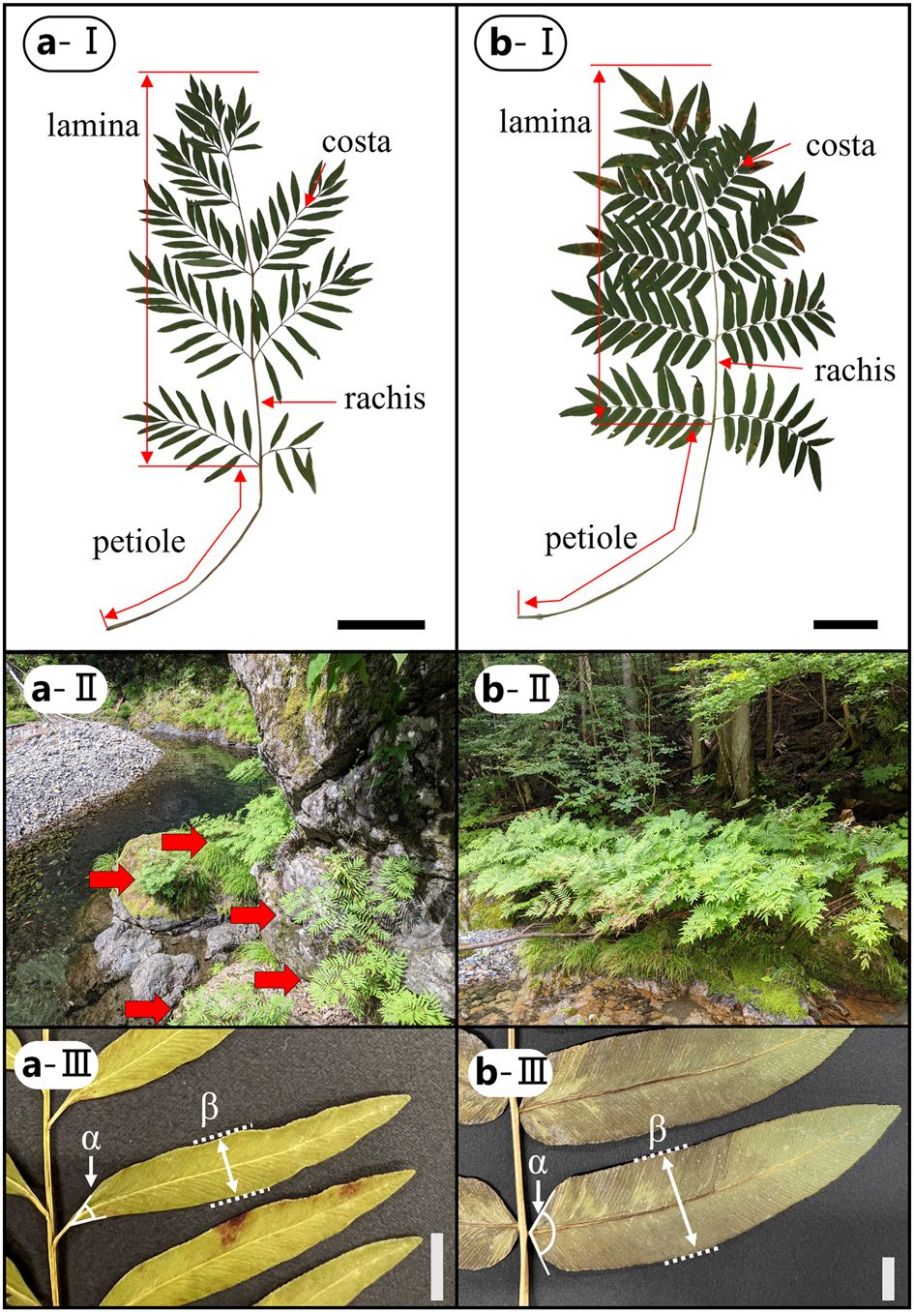
(**I**) Silhouettes of *O. lancea* (**a**) and *O. japonica* (**b**), bar = 10 cm; (**II**) Habitats of *O. lancea* (**a**) and *O. japonica* (**b**). Arrows indicate individuals of *O. lancea*; (**III**) Taxonomic characters between them. (α) angle at base of pinnules, (β) widest part of pinnules, bar = 10 mm. The measurement results for the angle at the base of pinnules and widest part of pinnules are shown in Table 1.

Among them, *O. lancea* Thunb. is an endemic plants along the river in Japan, capable of enduring the swift currents of rivers owing to its narrow lanceolate pinnules^7,13^ (Fig. 1). Comparative analysis of the morphology and anatomy of *O. lancea* and its closely related inland species *O. japonica* Thunb. revealed that the pinnule narrowing in *O. lancea* was attributed to a reduction in cell size along the width of the pinnules^13^. However, the structural and mechanical aspects of the petioles in *O. lancea* remain unknown.

Extensive research has been conducted in both agricultural crops and wood science, delving into the aspects of plant strength and flexibility, and their relevance in the context of construction applications^14–23^. Notably, recent studies have even extended to the model plant *Arabidopsis thaliana* (L.) Heynh, adding further insights to this area of investigation^24^. However, there have been very few studies that specifically examine the mechanical adaptations of plants to their distinctive environments^25–27^. Regarding stems, Niklas and Paolillo^28^ demonstrated that the mature epidermis plays a crucial role in reinforcing the parenchyma, underscoring the significance of mature outer tissues in stems as the principal structural components that support cells against tensile and bending loads. Therefore, to anatomically assess the strength and flexibility of *O. lancea* and *O. japonica*, we analyzed the cell size and cell wall composition of the subepidermal cortex of the petiole. Additionally, analyzing the mechanical properties related to petiole flexure, which are involved in the flexural modulus, flexural strength at break, and strain at break is essential in understanding their overall mechanical characteristics. In this test, a high bending strength indicates an enhanced fracture resistance, which serves as a critical determinant for ensuring the mechanical stability of the petiole when subjected to bending forces. In addition, the breaking strain, which shows the degree of deformation to breaking, is a factor where a higher value indicates that the petiole possesses a higher degree of flexibility. Therefore, employing these methodologies to compare the petioles of *O. lancea* and *O. japonica* has a potential to unveil novel insights into the adaptation of plant groups along the river, specifically concerning petioles, and to validate van Steenis’s hypothesis regarding adaptation of plant along the river. This study aims to clarify the mechanical properties of petioles in bending and the structural factors in the petiole cells of both species, while also discussing the anatomical differences observed in the petioles of the plant along the river.

## Methods

### Plant materials

In this study, wild populations of *O. lancea* and *O. japonica* were sampled from riparian (*O. lancea*: N35° 40′ 44.234″, E139° 04′ 25.068″) and inland (*O. japonica*: N35° 42′ 13.963″, E139° 00′ 53.744″) locations along the Tsuru River in Uenohara City, Yamanashi Prefecture, Japan (Fig. 1). Iwatsuki^29^ describes in Flora of Japan that *O. lancea* (Fig. 1a-I) and *O. japonica* (Fig. 1b-I) are distinguished by pinnules cuneate to acute (Fig. 1a-III) or subtruncate (Fig. 1b-III) at base, respectively, and the widest part of pinnules is less than 10 mm (Fig. 1a-III) for the former and 10–25 mm (Fig. 1b-III) for the latter. Therefore, we identified the samples collected according to this description (Fig. 1). This field research and sampling were carried out in areas where there were no collection restrictions under Japanese law. All methods were carried out in accordance with Japanese law. Voucher specimens are deposited in National Museum of Nature and Science (TNS). Following removal from the petiole base, the specimens were brought back to the laboratory on moistened newspapers to prevent water evaporation. Mechanical analyses were performed less than 12 h after of sample collection.

### Morphological analyses

We measured Angle at base of pinnules and Length at widest part of pinnules of *O. lancea* and *O. japonica* base on Iwatsuki^29^.

In the morphological analysis, lamina area was analyzed using the graphic software Fiji (ImageJ Version 1.53e). The shape of the petiole cross-section was assessed by measuring the long and short diameters using an electronic caliper. While this method is a simplified approach, it allowed for the calculation of the long to short diameter ratio, which was found to be approximately 1.1 for both species. Based on this observation, the transverse shape of the petiole was assumed to be elliptical. The petiole transverse area *A* was calculated using the following formula:

$$A = \frac{\pi ab}{4}$$

where *a* and *b* are the long and short diameters of the petiole, respectively. The relationship between the petiole transverse area *A* calculated by the above formula, and the lamina area supported by the petiole transverse area *A* was analyzed.

### Mechanical analyses

Due to the testing machine used in this study, a fixed span length of 50 mm was maintained for all conducted tests. Therefore, to apply the general bending theory, the petiole was selected to have a short diameter ranging from 3–4 mm. The samples were divided into sections approximately 60 mm in length, and the long and short diameters of the central portion of each sample were measured using calipers (CD-15CXR; Mitutoyo, Japan). These measurements were conducted to determine the transverse area of the petiole, following the same method employed in the morphological analyses. The span-depth ratio ranged from 13 to 16. To conduct the mechanical measurements, the same samples used in the anatomical measurements were subjected to a three-point bending test. This test was performed using a tabletop tensile and compression testing machine (MCT-1150, A&D, Japan) and an R5 bending test fixture (JM-B1-500N, A&D, Japan) with the test speed set to 10 mm/min.

Values of bending stress *σ* and strain *ε* were calculated using the following standard elliptical transverse formulas for the elastic loading of beams:

$$\sigma = \frac{M}{Z} = \frac{PL}{{4Z}}$$

and

$$\varepsilon = \frac{48\delta \sigma I}{{PL^{3} }} \times 100$$

where *M* is the bending moment of simply supported beams and *Z* is section modulus. Where *P* is the load, *L* is the span length, *δ* is the displacement, and *I* is the moment of inertia. In this study, strain *ε* is calculated as %.

The following formulae were used to calculate the section modulus *Z* and the area moment of inertia *I* of elliptical area:

$$Z = \frac{{\pi ab^{2} }}{32}$$

and

$$I = \frac{{\pi ab^{3} }}{64}.$$

In this study, the flexural stress and strain at the breaking point of the specimen were defined as the bending strength *σ*_max_ and the breaking strain *ε*_max_, respectively. The bending modulus of elasticity *E* was calculated as follows:

$$E = \frac{{\sigma_{0.25} - \sigma_{0.05} }}{{\epsilon_{0.25} - \epsilon_{0.05} }} \times 100.$$

Strain values from 0.05 to 0.25% and bending stresses were used to determine the bending modulus of elasticity. A sampling frequency of 50 Hz was used in this study. The testing machine used in this study was not sufficiently powerful to accurately measure the bending modulus of elasticity owing to the limited resolution of the strain measuring instrument. Nevertheless, the measurement of bending modulus of elasticity was conducted to determine potential alterations in the mechanical properties of supporting organs in plants along the river, which allow them to evade mechanical stresses caused by water currents.

### Measurement of cell wall area, cell area and cell length

After the mechanics test, petioles were air-dried in a draft chamber without heating and used to observe the petiole transverse tissue. The subepidermal cortex just below the epidermal layer on the abaxial side of the petiole cross-section was recorded using a tabletop electron microscope (TM3000, HITACHI, Japan), and analyzed using the ImageJ software. To determine the cell wall per unit area of the petiole, the cell wall area was measured within a 50 µm per side square section in the subepidermal cortex. As the cytoplasm, intracellular organelles, and intercellular spaces were compressed, in the dried petiole samples used, it was possible to measure only the cell wall. The cell wall fraction per unit area was calculated using the following formula:

$${\text{cell}}\;{\text{wall}}\;{\text{fraction}}\;{\text{per}}\;{\text{unit}}\;{\text{area}} = \frac{{\left( {{\text{cell}}\;{\text{wall}}\;{\text{area}}\;{\text{within}}\;2500\;{\upmu}\text{m}^{2} } \right)}}{{2500\;{\upmu}\text{m}^{2} }}.$$

Cell area was measured starting from cells in the subepidermal cortex adjacent to the epidermis. After measuring 10 cell area in the subepidermal cortex, the mean value was calculated and compared between the two species. The cell length along the vertical direction of petiole was measured using the maceration protocol of Gärtner and Schweingruber^30^. To macerate the tissue, a 0.1 mm thick razor (FA-10, Feather Safety Razor CO.,LTD, Japan) was used to cut the petiole longitudinally through the epidermis. Two types of cells were observed using the light microscopy (CX43, Olympus); one was the epidermis based on morphology, and the other was the sclerenchyma cell based on morphology. We measured the latter. The cell length was averaged by measuring 30 cells from one individual in the subepidermal cortex. Also, cells were stained with safranin to see if they were lignified. Base on Kijima^31^, a 5% safranin staining solution in 50% ethanol was prepared and maceration samples were stained on slide glass for 5 min, followed by thorough rinsing with water and observation of cells remaining on slide glass.

### Cell wall mass per unit volume

The various types of tissues were involved in the petiole and cell composition at the same position in petiole may be different among closely related species. Therefore, it was very difficult to compare the precise value of 'cell wall mass per unit volume'. However, 'cell wall mass per unit volume' played an important role to contribute the plant strength^28,32^. In this study, we selected 'cell wall mass per unit volume' to explain anatomical and mechanical results. The cell wall mass per unit volume is one of the factors that determine the mechanical properties of plants and is used as an indicator of the mechanical stability of stems. The volume *V*_*fresh*_ of a fresh petiole was calculated using the following formula:

$$V_{fresh} = \frac{\pi abl}{4}$$

where *l* represents the length of petiole. After volume measurements, the samples were dried in an incubator (DRM420DD, ADVANTEC, Japan) at 100 °C for 72 h, and the dry weight [g] was measured using an electronic balance (AG204, Mettler Toledo). The cell wall mass per unit volume in each petiole was calculated using the following formula:

$${\text{cell}}\;{\text{wall}}\;{\text{mass}}\;{\text{per}}\;{\text{unit}}\;{\text{volume}} = \frac{Dry\;weight}{{V_{fresh} }} .$$

### Statistical analysis

Microsoft Excel was used for statistical analysis. After obtaining equal variances for the comparison of each measured item (F-test), a t-test was performed under the assumption of “equal variances” or “variances not equal”. In this study, the F-test results indicated unequal variances in the width at widest part of pinnules, bending strength, bending modulus of elasticity, cell wall fraction per unit area, transverse cell area and cell length along the vertical direction of petiole. Equal variances were observed in the angle at base of pinnules and breaking strains.

## Results

### Morphological analyses

The angle at base of pinnules and width at widest part of pinnules were significantly sharper and shorter in *O. lancea*, respectively (Table 1).

**Table 1:** Measurements (mean ± standard error) of *O. lancea* and *O. japonica*.

Trait	*O. lancea*		*O. japonica*	
Morphological measurements				
Angle at base of pinnules (°)	52.6 ± 2.13	b	165.3 ± 3.16	a
Width at widest part of pinnules (mm)	8.0 ± 0.30	b	17.7 ± 0.99	a
Long/short diameter ratio	1.1 ± 0.01	a	1.1 ± 0.01	a
Mechanical measurements				
Bending strength *σ*_max_ (N/mm^2^)	34.9 ± 1.28	a	27.6 ± 2.19	b
Breaking strain *ε*_max_ (%)	7.7 ± 0.31	a	2.4 ± 0.20	b
Bending modulus of elasticity *E* (MPa)	1621.7 ± 71.48	a	1836.0 ± 200.02	a
Anatomical measurements				
Cell wall fraction per unit area	0.5 ± 0.01	b	0.6 ± 0.01	a
Transverse cell area (µm^2^)	127.5 ± 10.85	a	87.1 ± 4.11	b
Sclerenchyma length (µm)	723.2 ± 21.84	a	550.1 ± 31.26	b

Columns marked by different letters differ significantly according to the t-test (*p* < 0.05).

**Figure 2:**
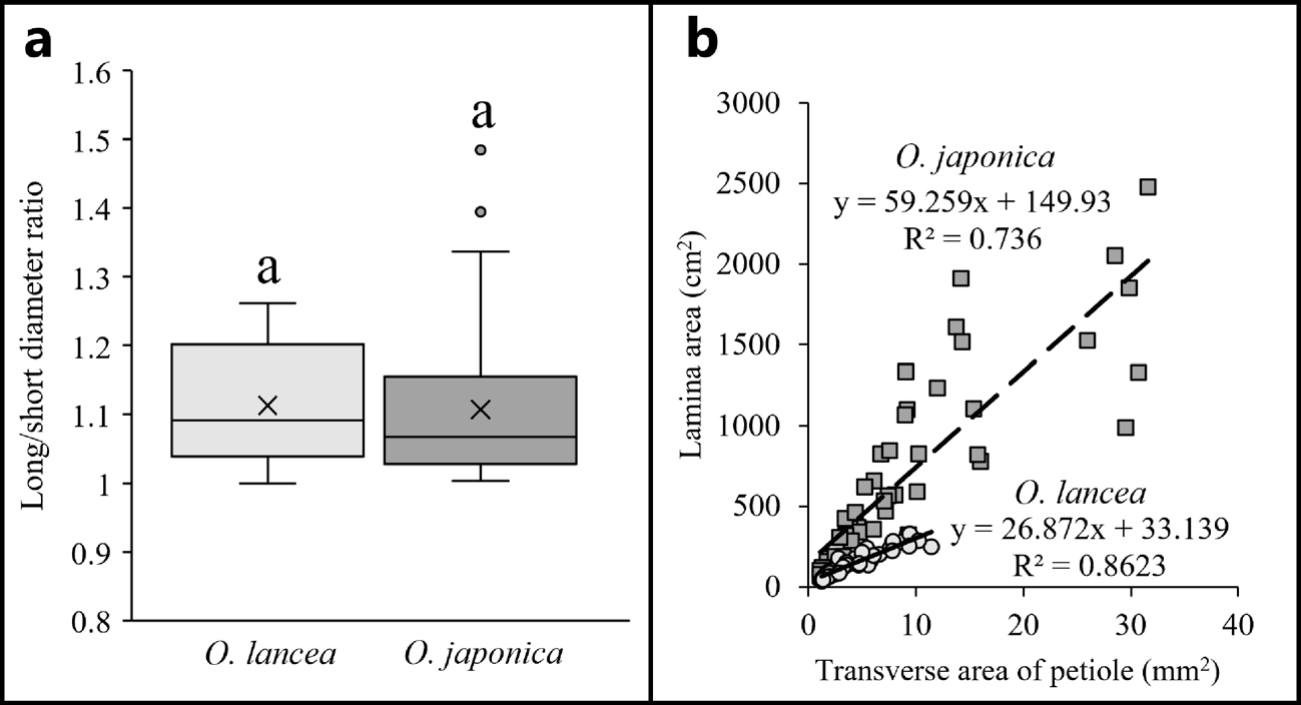
(**a**) Comparison of the long/short diameter ratio in the cross-section of petiole. Box whisker plots marked with identical letters were not significantly different according to t-test (*p* < 0.05). Crosses indicates position of the mean. (**b**) Relationship of transverse area of petiole and lamina area; circles indicate *O. lancea* and squares indicate *O. japonica*.

The long/short diameters ratio in petiole cross-sections of *O. lancea* and *O. japonica* showed no significant difference, indicating that both species had an elliptical transverse shape with the long/short diameter ratio of approximately 1.1 (Fig. 2a and Table 1).

An analysis was conducted on the relationship between the transverse area of the petiole and lamina area supported by the petiole transverse area in *O. lancea* and *O. japonica*. The results revealed a positive linear correlation between the transverse area of the petiole and lamina area. The data for *O. lancea* and *O. japonica* are plotted separately as distinct datasets in the same figure (N_*O. lancea*_ = 51, N_*O. japonica*_ = 81), and the regression lines are differentiated (Fig. 2b).

### Mechanical properties related to petiole bending

**Figure 3:**
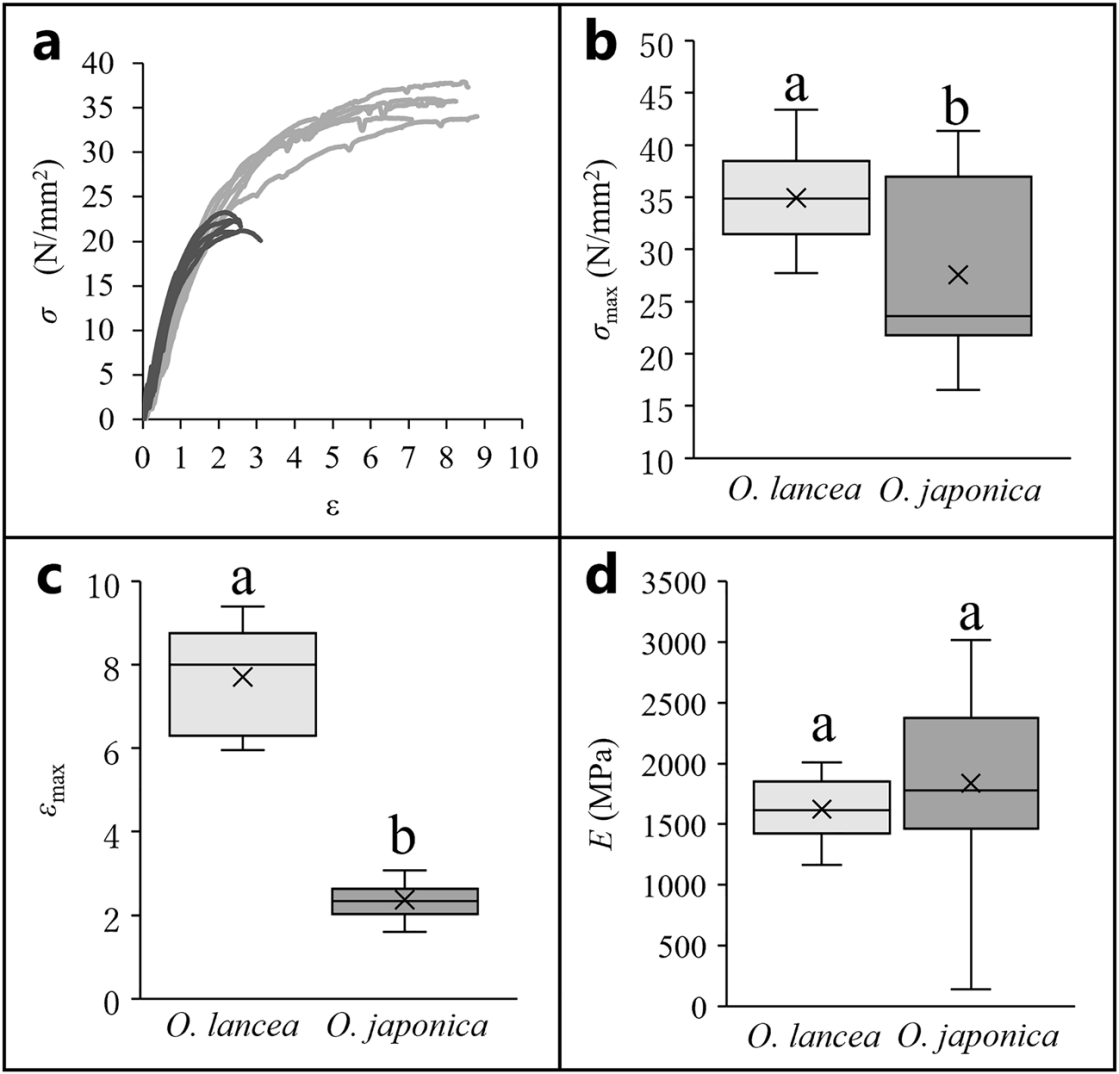
For mechanical analysis, (**a**) stress–strain curves are plotted; gray lines indicate *O. lancea* and black lines indicate *O. japonica*; five samples of each species are plotted; comparison of (**b**) bending strength and (**c**) breaking strain, (**d**) bending modulus of elasticity in the petiole. Box whisker plots marked with different letters differ significantly according to t-test (*p* < 0.05). Crosses indicate position of the mean.

When comparing the mechanical properties of petioles, we found that the bending strength *σ*_max_ and breaking strain *ε*_max_ of *O. lancea* were significantly greater than those of *O. japonica*. Although no significant difference in the bending modulus of elasticity, *E*, was observed, the mean value was greater for *O. japonica* than for *O. lancea* (Fig. 3 and Table 1).

### Cell wall fraction per unit area, transverse cell area and sclerenchyma length along the vertical direction in subepidermal cortex of petiole

**Figure 4:**
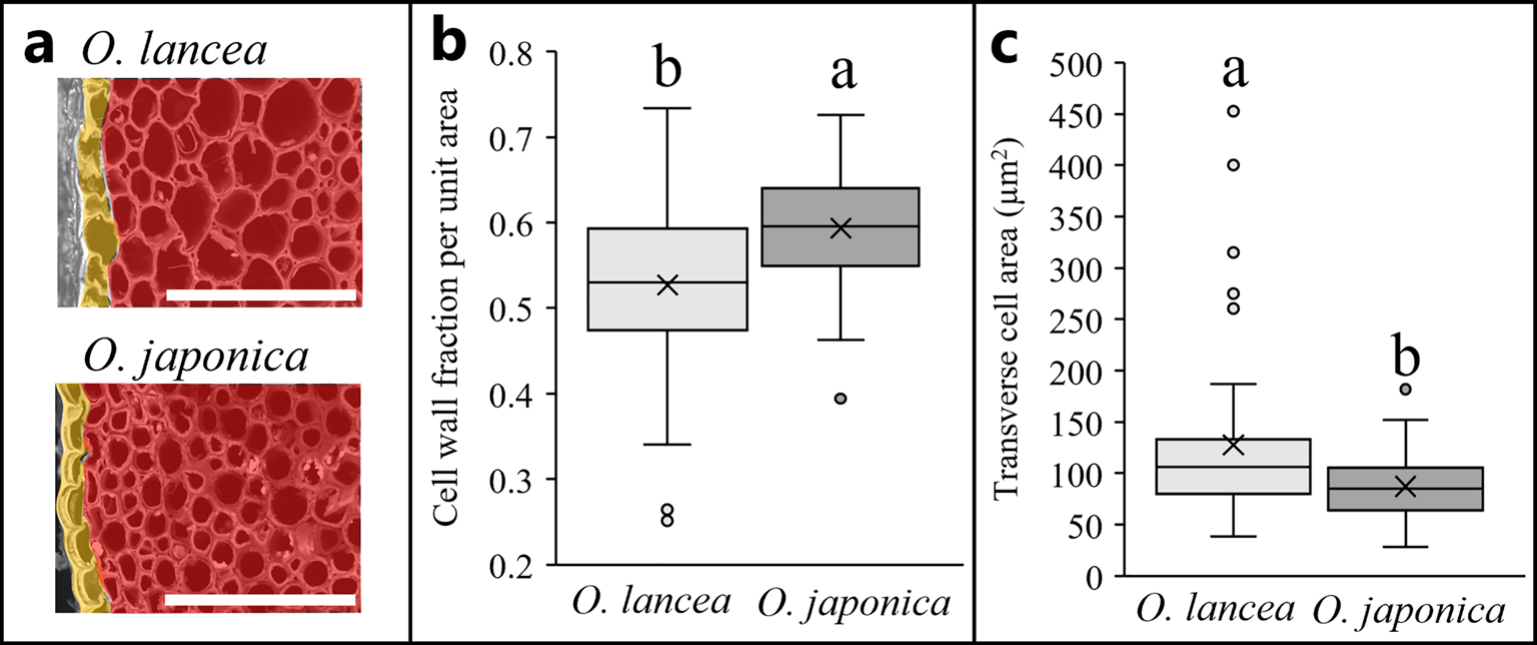
For anatomical analysis, (**a**) SEM image of petiole cross-section in the subepidermal cortex; bar = 100 µm; (**b**) comparison of cell wall fraction per unit area; (**c**) transverse cell area in the petiole. Epidermis and subepidermal cortex indicate yellow and red, respectively. Box whisker plots marked with different letters differ significantly according to t-test (*p* < 0.05). Crosses indicate position of the mean.

A comparison of the cell wall fraction per unit area in the petiole cross-section indicated that *O. lancea* has a significantly smaller cell wall fraction in the subepidermal cortex than *O. japonica*. The transverse cell size of the subepidermal cortex was significantly larger in *O. lancea* than in *O. japonica* (Fig. 4 and Table 1).

**Figure 5:**
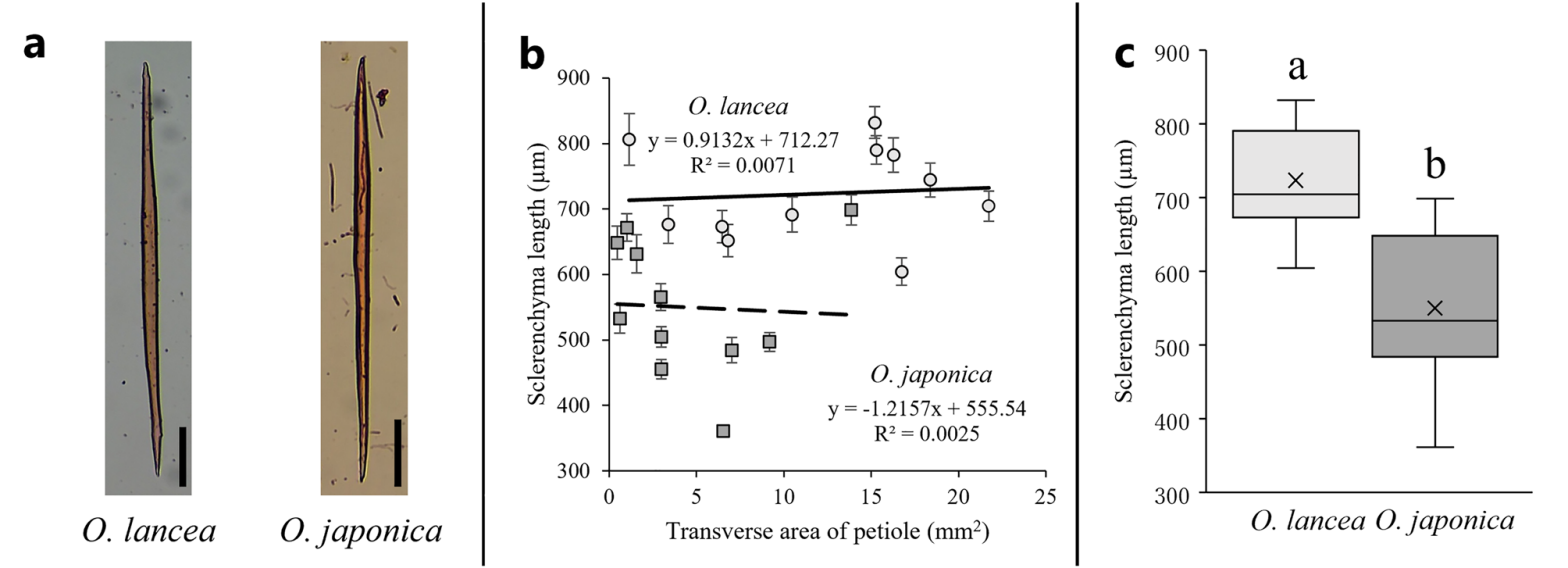
Sclerenchyma data, (**a**) safranin-stained cortex sclerenchyma in lateral view, bar = 100 µm; (**b**) relationship of transverse area of petiole and sclerenchyma length; circles indicate *O. lancea*, squares indicate *O. japonica*; (**c**) comparison of sclerenchyma length. Box whisker plots marked with different letters differ significantly according to t-test (*p* < 0.05). Crosses indicate position of the mean.

The cells along the vertical direction were confirmed to be lignified from safranin staining (Fig. 5a). For sclerenchyma length, the relationship between petiole transverse area and sclerenchyma length indicated that sclerenchyma length was almost constant with respect to the transverse area (Fig. 5b). The results of measurements of sclerenchyma length in the subepidermal cortex indicated that *O. lancea* was significantly longer than *O. japonica* (N_*O. lancea*_ = 11, N_*O. japonica*_ = 11: Fig. 5c and Table 1).

### Cell wall mass per unit volume of petiole

**Figure 6:**
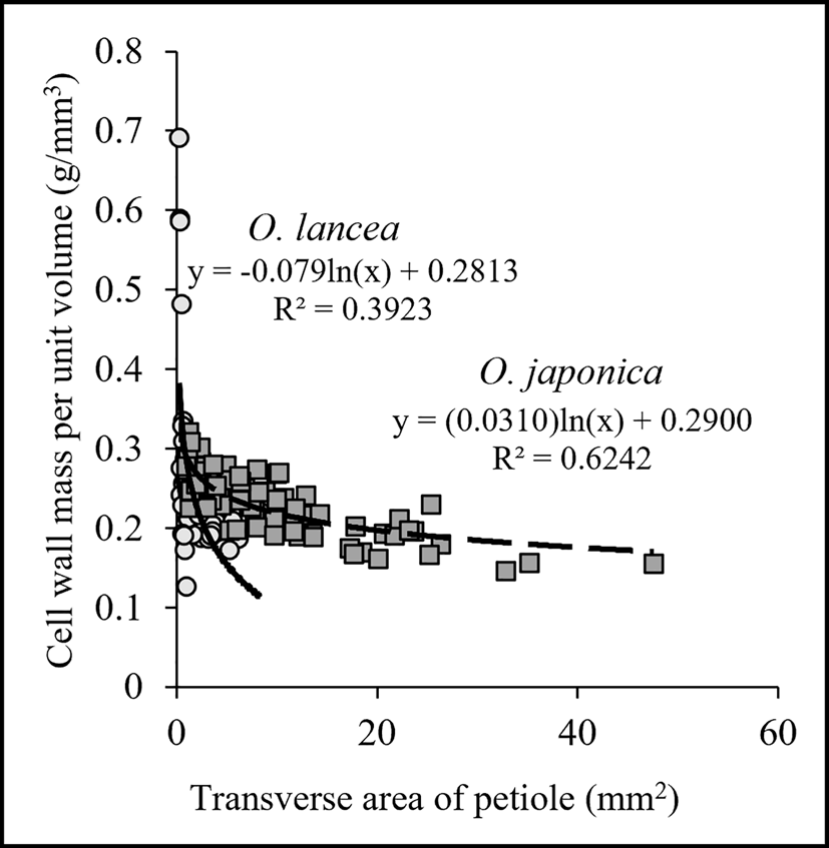
Relationship of cell wall mass per unit volume and transverse area of petiole; circles indicate *O. lancea*, squares indicate *O. japonica*; the log-approximation curve is illustrated.

In Fig. 6, the transverse area of petiole is plotted against the cell wall mass per unit volume. Data for *O. lancea* and *O. japonica* are plotted in separate datasets within the same Figure (N_*O. lancea*_ = 54, N_*O. japonica*_ = 82), and the logarithmic approximations are differentiated. The transverse area of petiole revealed a negative curvilinear correlation with cell wall mass per unit volume.

## Discussion

In terms of mechanical properties, our study found that the bending modulus of elasticity was similar between *O. lancea* and *O. japonica* but there were significant differences in the bending strength and breaking strain, indicating that the petiole of *O. lancea* was more resistant to fracture and displayed greater flexibility until the point of breakage (Fig. 3). To gain insights into the underlying mechanism behind these mechanical differences between the two species, our initial focus was on conducting morphological analyses of the transverse shapes of their petioles. The petioles of both species exhibited similar elliptical transverse shapes, implying that the shape did not account for the contrasting mechanical properties observed in the two species. Considering the anatomical point of view, Shah et al.^32^ proposed that the quantity of cell walls contributes to the strength of an organ, implying that the variation in the cell wall content between organs may influence their mechanical properties. Thus, in our subsequent analysis, we compared the cellular structure in the subepidermal cortex and observed that the cell wall fraction per unit area in *O. japonica* was significantly higher than that in *O. lancea*. Moreover, this anatomical analysis indicated that the cell area in the horizontal section of *O. lancea* was significantly larger than that of *O. japonica*. In addition, sclerenchyma length along the vertical direction of petiole in the subepidermal cortex was longer in *O. lancea* than in *O. japonica*. For the cells observed in this study, lignification was confirmed by safranin staining. This result supports the lignification of the epidermal layer's underlying tissue in the petiole of young leaves reported by Imaichi and Kato^12^. The relationship between petiole transverse area and cell wall mass per unit volume indicated that the cell wall mass per unit volume tended to decrease greatly as petiole transverse area increased in *O. lancea* compared to *O. japonica*. These results indicated that the cell volume in the subepidermal cortex of *O. lancea* may be larger both horizontally and vertically compared to *O. japonica.* Imaichi and Kato^14^ reported that the cell area in parenchyma was smaller in *O. lancea* than in *O. japonica*. For epidermal cells, the cell wall of *O. japonica* is thicker than that of *O. lancea*^12^. However, with respect to the subepidermal layer just below the epidermal layer, it has not been mentioned which has a larger cell area or cell wall thickness. Therefore, the results of cell size in the subepidermal cortex of the petiole of *O. lancea* represent a new finding. How does a larger cell volume in the petiole of *O. lancea* contribute to an increased strain capacity? We were hypothesized that one of the reasons for this could be the increased span length per cell, which creates a greater distance between the fulcrums and allows for a higher strain capacity in the petiole. However, as the cell volume increases the number of cell walls per petiole decreases, which directly impacts the mechanical properties of the petiole. Therefore, *O. lancea* achieves flexibility through petioles with increased cell volume. To our knowledge, this study is the first to provide insights into the mechanical characteristics of petioles in plant groups along the river. van Steenis^7^ called plant groups along the river "rheophytes" and suggested that rheophytes have acquired tough and flexible petioles, stems, and branches to reduce or avoid water flow stress caused by sudden increases in water level. Our findings support the hypothesis proposed by van Steenis^7^, thus validating its relevance. Some plant groups with narrow leaves along the river have been reported for rheophyte in various phylogenetic lineages^33–42^, suggesting that these species have flexible stems, branches, and petioles. We believed that our results were one of evidence to construct the flexible petiole by changing the cell volume involving in the low cell wall fraction per unit area of subepidermal cortex and relatively long sclerenchyma cells. However, it is difficult to fully explain the mechanical properties of the entire petiole with only the anatomical analysis of this study, and it needed to conduct future analysis of the structure and morphology of other tissues. In addition, although our mechanical results could discuss the petiole of *O. lancea* avoiding water currents, it needs to discuss the petiole involving in the load that lamina receives in the future. Also, petioles in water streams are subjected to torsion and tension in addition to bending. Therefore, assessing and comparing other mechanical properties, such as torsion and tensile tests, provide multifaceted insight into the avoidance mechanisms of *O. lancea* in water streams.

In general, plants increase the number of leaves to acquire more resources through photosynthesis as they grow^43^. The results of this study indicated that there is a large difference in the pinnule area at transverse area of petioles between *O. lancea* and *O. japonica*. Support organs, such as stems and petioles may bend under the weight of laminas and pinnules, leading to a reduction in bending resistance, commonly referred to as the Brazier effect^32,44^. As the pinnule develops, it is essential to enhance the strength of the petiole. However, in the case of *O. lancea*, the acquisition of displacement in the petiole hinders the increase in pinnule area by reducing the cell wall content, which is crucial for petiole strength. This suggests that lamina area in *O. lancea* is reduced in exchange for greater petiole displacement. However, the relatively small lamina area in *O. lancea* may serve to mitigate the impact of water flow on the petiole. This property is particularly significant for *O. lancea*, as it is a species that experiences submersion in rivers during sudden floods and rising water levels. Moreover, our results indicated that the bending strength of *O. lancea* was significantly higher than that of *O. japonica*, suggesting that the petiole of the former was less likely to break than that of the latter when submerged. The results revealed that while *O. lancea* exhibited the ability to thrive in environments where many other species struggle to establish themselves, it faced limitations in colonizing areas where other species prevail. This constraint can be attributed to the small area of pinnules in *O. lancea*, which corresponds to a lower amount of cell walls.

Recently, dam construction and river modifications that alter the riparian environment have been shown to disrupt the specific water flow stresses of river sections^45^, potentially allowing the invasion of competitor species into the habitat of *O. lancea* and leading to a decline in their population size. Mitigating the occurrences of flooding and rising water levels along the river is beneficial for neighboring communities. However, from the perspective of *O. lancea* this poses a challenge as it could potentially lead to the expulsion or disappearance of this species from its natural habitat.

## Data Availability

The datasets generated during and/or analyzed during the current study are available from the corresponding author on reasonable request.
